# New Appliances and Things Medical

**Published:** 1897-10-16

**Authors:** 


### NEW APPLIANCES AND THINGS MEDICAL.

[We shall be glad to receive, at our Office, 28 & 29, Southampton Street, Strand, London, W.C., from the manufacturers, specimens of all new preparations and appliances which may be brought out from time to time.]

### NEW PREPARATIONS.

(Lorimer and Co., Brittannia Row, Islington.)

From among a large number of excellent preparations submitted to us by the above firm we select a few for description. In the first place the St. John's first aid lotion and embrocation seems a useful household remedy for bruises, cuts, and surface abrasions. This lotion is a remarkably clean preparation, and appears to have some antiseptic properties, in addition to an astringent and stimulating action. Chocolat aperiens is a highly ingenious medium for the pleasant administration of an aperient to refractory or delicate children. The small sticks differ neither as regards appearance or taste from ordinary chocolate, and two small sticks will prove a sufficient dose for a child of four years. Other members of the St. John's series are the germicide soap, a preparation of excellent quality both from the point of view of antisepsis and as a toilet article—useful alike for the surgeon and for those coming in contact with infectious cases. Another example is the St. John's headache cure, bearing a name, however, of questionable propriety. It is a combination of quinine and antipyrine, dispensed in cachets. To keep pace with public demands Messrs. Lorimer and Co. can show some very good examples of coca wine and beef extract, the latter manufactured to their order on Liebig's principle. So far as we have been able to examine the various preparations of this firm they appear to us to be of excellent quality, and good specimens of latter-day pharmacy.

### MAGGI'S CONSOMMÉ FOR HOSPITAL USE.

(London Agents: Messrs. Cosenza and Co., 95, Wigmore Street.)

In our issue of September 26th, 1896, we drew attention to Maggi's Concentrated Consommé. It was then a novelty to the public in this country, but we prophesied a future for it, and to-day there is no more popular invalid specialité. We are, therefore, glad to learn that the manufacturers are selling it in bulk for hospital use. It is prepared in blocks of 1 lb., divided up into sections. For hospital use a section containing 12 portions should be dissolved in nine pints of perfectly boiling water. This will make a capital and appetising clear soup of the very best description at a cost of very little more than one shilling—that is to say, less than a penny per cup of half a pint. It is undoubtedly the cheapest concentrated soup on the market, and for those of fastidious taste, in our opinion, the best. As a nitrogenous food it cannot, of course, compare with many of the concentrated meat extracts on the market; but these are in many cases distinctly unpalatable to the invalid. Maggi's Consommé is never so, and in our experience we have found it remarkably reviving, considering the small percentage of solids contained in the portion necessary for one person.

### INVALID FURNITURE.

(Messrs. Farmer, Lane, and Co., 77 and 79, New Oxford Street.)

Medical men and hospitals managers alike will appreciate the variety and excellence of the appliances for the sick-room shown by Messrs. Farmer, Lane, and Co., 77 and 79, New Oxford Street. Illness is made more endurable and the lot of the sufferer brightened by the facilities afforded him of movement without undue exertion. The variety in chairs is enormous. There are carrying chairs, bath chairs, reclining chairs, and self-propelling chairs. Of these perhaps the two latter deserve special mention, as being not only convenient but most comfortable for delicate and helpless people. The self-propelling Merlin chair is most luxurious. The frame is of well-polished wood, caned, and finished off with cushions as preferred. The back and leg rest is adjustable to any angle, and the arms are moveable with the back. It will thus be seen that it can be made to serve the twofold purpose of a couch or chair as required. A cheap carrying chair made in a combination of cane and bamboo is very light and inexpensive, only costing 12s. 6d. There is also an ingenious folding chair, which serves a variety of purposes, being capable of many metamorphoses. The Ilkley couch seems a useful contrivance, and can be adjusted at any angle. Messrs. Farmer, Lane, and Co. have just brought out an improved couch of this description with arm rests that are adaptable with the rise of the back, it also can be converted into a comfortable easy chair, the leg-rest sliding under the seat when no longer required. Most of these couches have the advantage of folding up flat for travelling. There is a delightful form of lounge made in light cane, to which we desire to draw special attention. In shape somewhat like a deck chair, it has an adjusting mechanism by which it becomes flat and makes an excellent temporary bed in case of illness. Reading stands and reading lamps are another speciality of this enterprising firms.

The commode chairs are a great improvement on the old-fashioned article hitherto commonly in vogue. When not in use the pan can be removed, and a comfortable armchair remains.

### SOL. IODI (DOWNES).

(Agents: Brooks and Co., 136, Lower Baggot Street, Dublin.)

This is a comparatively new preparation of iodine, which has been invented and prepared by Mr. R. T. Downes, of Dublin. The value of iodine as an external application is admittedly high, but its sphere of usefulness is often limited by the unsightly discoloration of the skin which invariably follows its application. This new preparation does not impart a colour to the skin, while in our experience the therapeutic results are efficacious. Sol. iodi may be applied to the part affected, and thoroughly rubbed in, so as to ensure absorption.

### CONDENSED MILK, "PICNIC" BRAND.

(Condensed Milk Company, Holland, Limited, Rotterdam).

The "Picnic" brand of condensed milk, which is prepared by the Condensed Milk Company of Holland (Limited), Rotterdam, is a good example of a pure condensed milk to which sugar has been added for preservative purposes. According to the analysis of the sample sent to us it contained 73·8 per cent. dry milk solids and sugar, with only 26·2 per cent. of water. It was satisfactory to find a good proportion of fat, namely, nearly 8 per cent., as many brands of condensed milk are deficient in this important ingredient. As a saccharine condensed milk, the "Picnic" brand as submitted to us can be used with advantage.

